# Fruit Quality Assessment of Novel Hybrid Pummelo × Sweet Orange and Its Molecular Characterization Using Acidity Specific Markers

**DOI:** 10.17113/ftb.62.01.24.8349

**Published:** 2024-03

**Authors:** Raushan Kumar, Nimisha Sharma, Anil Kumar Dubey, Radha Mohan Sharma, Shruti Sethi, Gyan Prakash Mishra, Sandeep Mathur, Hatkari Vittal, Mukesh Shivran, Neha Sharma

**Affiliations:** 1 Division of Fruits and Horticultural Technology, ICAR-Indian Agricultural Research Institute, Pusa Campus, 110012 New Delhi, Delhi, India; 2 Division of Food Science and Postharvest Technology, ICAR-Indian Agricultural Research Institute Pusa Campus,110012 New Delhi, Delhi, India; 3 Division of Seed Science and Technology, ICAR-Indian Agricultural Research Institute, Pusa Campus, 110012 New Delhi, Delhi, India; 4 ICAR-National Institute for Plant Biotechnology, Pusa Campus, 110012 New Delhi, Delhi, India

**Keywords:** acidity specific markers, ascorbic acid, antioxidants, nutritional quality, hybrids, pummelo, sweet orange

## Abstract

**Research background:**

There is considerable diversity in newly developed pummelo × sweet orange citrus hybrids. Most hybrids showed lower peel thickness and high juice yield but there is a lack of information on fruit quality parameters and molecular characterization. Therefore, the aim of the current study is to determine the content of antioxidants and properties of the fresh juice of 24 new pummelo × sweet orange citrus hybrids (*Citrus maxima* [Burm. f.] Osbeck × *Citrus sinensis* [L.] Osbeck) and the parental genotypes along with molecular characteristics determined using acidity specific markers.

**Experimental approach:**

The correlation and estimate of inheritance of the fruit juice properties: ascorbic acid, total phenol, total flavonoid, total antioxidant, total soluble solid and sugar contents, pH, titratable acidity, along with sensory evaluation was performed. Molecular characterization of these hybrids was carried out using *de novo* generated acidity specific simple sequence repeat (SSR) markers.

**Results and conclusions:**

The main constituents of the fruit juice of pummelo × sweet orange hybrids were observed in the range of *w*(ascorbic acid)=40.00–58.13 mg/100 g, total phenols expressed as gallic acid equivalents *w*(GAE)=40.67–107.33 mg/100 g, total antioxidants expressed as Trolox equivalents *b*(Trolox)=2.03–5.49 µmol/g, total flavonoids expressed as quercetin equivalents *w*(QE)=23.67–59.33 mg/100 g, along with other properties: total soluble solids=7.33–11.33 %, *w*(total sugar)=2.10–5.76 %, *w*(reducing sugar)=1.69–2.78 %, *w*(non-reducing sugar)=0.39–3.17 % and titratable acidity 1.00–2.11 %. The above parameters differed significantly in the fruit juice of the evaluated pummelo × sweet orange hybrids. Considering these parameters, the hybrids SCSH 17-9, SCSH 13-13, SCSH 11-15 and SCSH 3-15 had superior antioxidant properties in terms of these parameters. A higher heritability (≥80 %) was also observed for all juice properties. Molecular characterization of pummelo × sweet orange hybrids showed that >50 % of the hybrids were grouped with medium acidity parents. Both molecular and biochemical parameter-based clustering showed that interspecific hybrids exhibit transgressive segregation with increased antioxidants that help alleviate the health problems.

**Novelty and scientific contribution:**

These newly developed pummelo × sweet orange citrus hybrids are a valuable source of high-quality antioxidants for a healthy diet. The identification of trait markers that enable selection at the seedling stage is of great benefit to citrus breeders, as the characteristic features of a mature tree are not yet visible at the juvenile stage.

## INTRODUCTION

Malnutrition affects over two billion people worldwide, with 924 million experiencing undernourishment. This condition contributes to approx. 45 % of deaths among children under the age of five (*1*). Around 828 million people worldwide suffer from hunger, and the outbreak of the Covid-19 pandemic has further worsened the situation (*2*). The highest number of undernourished people (15.2 %) live in India and about 38.4 % of children (<5 years) in India are stunted, while more than 35.7 % are underweight (*3*). Zero hunger and good health are the most important goals of the United Nations. The Sustainable Development Goals 2023 Report has shown how wide gaps are in achieving the targeted goals. Citrus fruits are widely cultivated across the globe and are considered the most commonly consumed fruits that promote health (*4*). Due to their pharmaceutical properties, including anti-inflammatory, antisclerotic, antiviral, antibacterial and anticancer properties, citrus fruits have long been valued as an essential part of a healthy diet (*5*). Despite being rich in nutrients, the nutritional properties of citrus fruits have often gone unnoticed (*6*, *7*). Various species of citrus fruits have different chemical compositions. The main components of the sweet orange include sugars (such as glucose, fructose and sucrose) and acids, mainly citric acid and a smaller amount of malic acid. Citrus fruits contain a considerable amount of ascorbic acid (*8*). In most citrus fruits, the total soluble solids (TSS) in the fruit juice typically range from 8–12 %, while the titratable acidity (TA) is commonly within the range of 0.5–1.5 %. Generally, oranges and mandarins have a TSS/TA ratio of 12–14:1, but in pummelo it is 6:1. The vitamin C in the fruit juices ranged between 25 and 85 mg per 100 g of juice. Citrus fruits are also rich in nutrients such as flavonoids and fibre, which play a role in protecting arteries, reducing inflammation, improving gastrointestinal health and potentially preventing diseases such as diabetes, cancer and neurological disorders. Vitamin C is essential for the development and maintenance of strong bones, skin, connective tissues and blood vessels. The immune system is strongly supported by vitamin C, which also functions as an antioxidant that can protect cells from the damaging effects of free radicals and helps to reduce inflammation (*9*). The non-haem iron found in plant foods is absorbed by the body with the help of vitamin C. Eating citrus fruits in combination with plant foods like nuts, seeds and legumes therefore helps to improve the body’s absorption of iron. The preventive properties of citrus fruits extend to a wide range of nutrients, including a large family of plant chemicals called flavonoid (*10*). The bioactive chemical compounds found in citrus fruits, such as flavonoids, carotenoids, terpenes and limonoids, have promising potential in combating obesity, inflammatory conditions, atherosclerosis, neurological disorders and cancer due to their remarkable antioxidant properties. By reducing fat absorption, pancreatic lipase (PL), an essential enzyme involved in the hydrolysis of triglycerides in the digestive system, can reduce obesity. There is a constant demand for the development of new improved cultivars fortified with minerals.

Hybridization is an important method for the improvement of perennial fruit plants, especially citrus fruits, enabling advances in tree growth characteristics, longer harvest of high-quality fruits and imparting resistance to both biotic and abiotic stresses (*11*). In contemporary citrus crop improvement programs, the focus is on developing high-quality fruits enriched with health-promoting bioactive compounds as the targeted traits (*11*). Pummelo (*Citrus maxima* (Burm. f.) Osbeck) is an important citrus species known for its health-promoting properties. Nevertheless, it faces challenges in fruit quality, characterized by a thick peel, a lower juice amount and a higher seed count. For that reason, a systematic citrus improvement programme was launched at the beginning of the 21st century at ICAR-Indian Agricultural Research Institute, New Delhi, India. The main objective of this programme was to improve the quality of pummelo through strategic crossbreeding with sweet orange. Currently, there is no record of a citrus variety specifically developed for high chemical nutrient content in the fruit juice. Considering the widespread problem of malnutrition, the cultivation of nutrient-rich citrus varieties promises to significantly reduce disorders related to mineral nutrition in humans. A critical prerequisite for this endeavour is understanding the composition of the fruit to identify hybrids with superior nutritional values. The characterization of different hybrids and parents plays a crucial role in the breeding of elite cultivars. Considering these circumstances, the present study was conducted to evaluate the concentration of phytochemical nutrients in interspecific citrus hybrids, particularly in orangelo, comprising both parental genotypes. The study also aims to determine correlations between juice nutrients, estimate genetic variability in citrus fruit juice and characterize these hybrids using recently developed acidity-specific SSR markers.

## MATERIALS AND METHODS

### Experimental site and plant materials

The current study was carried out during 2022–2023 on a group of 24 orangelo hybrids, aged between 7 and 10 years developed at ICAR-Indian Agricultural Research Institute, New Delhi. These hybrids were derived from four parental genotypes of pummelo (*C*. *maxima* [Burm. f.] Osbeck) and sweet orange (*Citrus sinensis* [L.] Osbeck), which were evaluated for the content of nutrients in fruit juices. The specific hybrid genotypes and their parentage information can be found in Table S1. The hybrid seedlings were planted at a distance of 3 m×3 m, while the parental genotypes were budded onto *Citrus jambhiri* Lush. rootstock. The experimental site is located in a typical subtropical climate characterized by hot and dry summers followed by cold winters. All plants were irrigated and fertilized uniformly, following the recommended practices for growing citrus plants in the same agro-climatic zone. Recommended production techniques were applied to all hybrids, including the parents.

Mature fruits were harvested from different parts of the tree at proper maturity as determined by the standardized ratio of total soluble solids (TSS) and titratable acidity (TA) for each genotype from the main orchard of the Fruits and Horticultural Technology Division, ICAR-IARI, New Delhi, India.

After harvesting, each fruit of pummelo × sweet orange hybrids and their parents were washed thoroughly under tap water and dried with a paper towel to remove surface impurities. Each fruit was cut in half and the juice was extracted with the citrus press juicer and filtered. The juice yield was calculated and stored at –20 °C until analysis.

### Measurement of juice recovery, pH, TSS, TA and TSS/TA ratio

The juice recovery from each of the selected citrus fruits and the overall juice recovery (%) was calculated using the following formula:


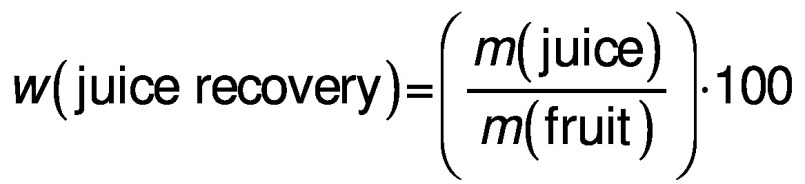
 /1/

The pH of the juice was analyzed using digital pH meter (pH meter 700; Eutech Instruments, Vernon Hills, NY, USA). The TSS was measured using digital refractometer (MA871; Milwaukee Instruments, Inc., Rocky Mount, NC, USA). The values were expressed in %. The machine was standardized using purified water before readings.

The TA was determined as percentage using AOAC method no. 947.05 (*12*). A volume of 10 mL of each juice was measured into a volumetric flask and then made up to 100 mL mark using distilled water. Then, 10 mL of each diluted juice was titrated with 0.1 M NaOH using phenolphthalein (Indenta Chemicals, Mumbai, India) as an indicator until a pale pink colour developed. The titratable acidity (%) was then calculated as citric acid as follows:


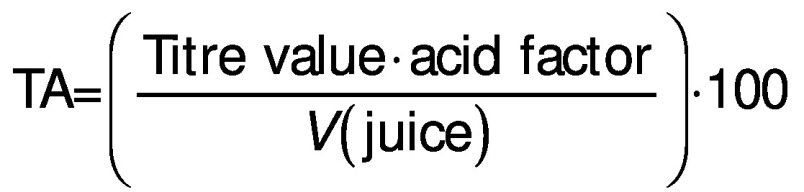
 /2/

where acid factor for citric acid was 0.0064 and *V*(juice)=10 mL. The TSS/TA ratio was calculated from the obtained data.

### Determination of ascorbic acid, total flavonoid content, total phenolics, total antioxidant activity and sugar mass fractions

The ascorbic acid concentration was determined using AOAC method no. 967.21 (*13*). A volume of 10 mL of citrus juice was taken and filled up to 100 mL with 3 % metaphosphoric acid (Loba Chemie Pvt. Ltd, Mumbai, India). Then, a 10-mL aliquot of the metaphosphoric extract was taken and titrated with the standard solution of 2,6-dichlorophenol-indophenol dye (HiMedia Laboratories, Pvt. Ltd., Mumbai, India) to the titration endpoint and the development of pink colour which should persist for 15 s. The ascorbic acid mass fraction in mg/100 g was calculated using the following equation (*14*):


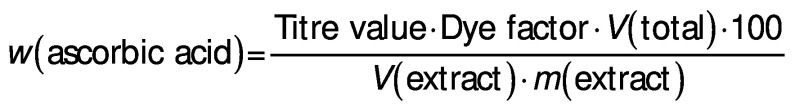
 /3/

where dye factor (mg/mL) was 0.5 mg/titre value (mL).

The total flavonoid content of the citrus fruits was estimated by a calorimetric method using the aluminium chloride reagent (Sigma-Aldrich, Merck, Burlington, MA, USA) and quercetin as standard (*15*). A volume of 1 mL of citrus juice was extracted in 10 mL of 80 % ethanol (SLC Chemicals, Patparganj, Delhi, India). Then, the mixture was centrifuged (Sigma Zentrifugen, Osterode am Harz, Germany) at 10 000×*g* and 4 °C for 20 min. A volume of 1 mL of properly diluted extract (*γ*=1 mg/mL) was mixed with 1.4 mL of distilled water and 0.3 mL NaNO_2_ (5 % *m*/*V*). Additionally, 0.3 mL of AlCl_3_ (10 % *m*/*V*) was added 5 min later and allowed to react for another 6 min. After that, 2 mL of 1 M NaOH solution were added. The resultant mixture was then made up to a total volume (*V*) of 5 mL using distilled water. The solution was thoroughly mixed and its absorbance (*A*) was measured using a UV-Vis spectrophotometer (GENESYS; Thermo Fisher Scientific, Waltham, MA, USA) at a wavelength of 510 nm. The total flavonoid content (TFC) was expressed in mg of quercetin equivalents (QE) per 100 g according to the following equation:


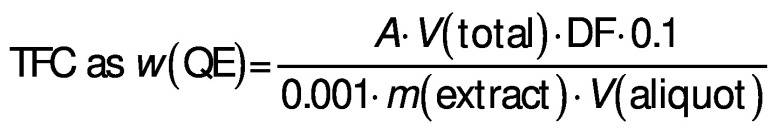
 /4/

where DF is dilution factor and 0.001 is QE factor.

Total phenolic content of each citrus juice extract was determined using the Folin-Ciocalteu colorimetric method suggested by Singleton *et al*. (*16*). A mass of 1 g of citrus pulp was crushed in a mortar with pestle using 80 % ethanol and centrifuged (Sigma Zentrifugen) at 10 000×*g* for 20 min. To 100 µL supernatant, 2.9 mL distilled water were added along with 0.5 mL 0.2 M Folin-Ciocalteu reagent (Sigma-Aldrich, Merck). After resting for 10 min at room temperature, 2 mL of sodium carbonate solution (20 % *m*/*V*) were added. The solutions were mixed and allowed to incubate at room temperature for 30 min. The absorbance (*A*) of dark blue coloured complex was measured with a UV-Vis spectrophotometer (GENESYS; Thermo Fisher Scientific) at 760 nm. Total phenolic content (TPC) was expressed in mg of gallic acid equivalents (GAE) per 100 g of dry extract according to the following equation


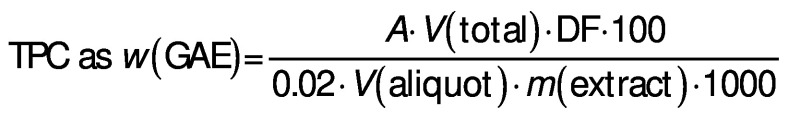
 /5/

where 0.02 is GAE factor.

The DPPH radical scavenging capacity of each citrus juice extract was determined as suggested by Brand-Williams *et al.* (*17*) with some modifications. A solution of 0.06 mM 2,2-diphenyl-2-picryl-hydrazyl (DPPH) in methanol (Sigma-Aldrich, Merck) was prepared and 2.9 mL of this solution were mixed with 0.1 mL of each extract previously dissolved in methanol. The mixture was kept in the dark at room temperature for 30 min. The absorbance (*A*) was measured using a UV-Vis spectrophotometer (GENESYS; Thermo Fisher Scientific) at 517 nm, and inhibition of juice was calculated as:


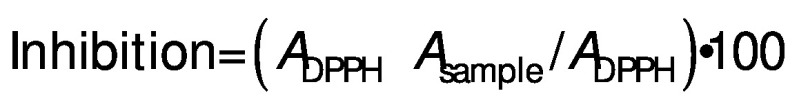
 /6/

From the results, DPPH radical scavenging capacity was calculated as *b*(Trolox) in µmol/g according to the following equation:


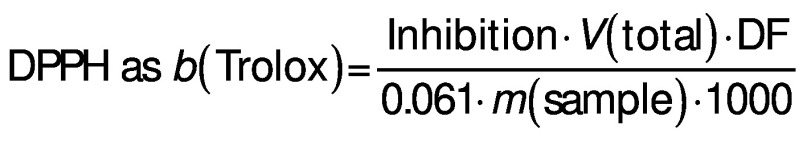
 /7/

where DF is dilution factor, *V*(total)=3 mL and 0.061 is DPPH standard solution absorbance.

Total sugars (%), reducing sugars (%) and non-reducing sugars (%) were estimated according to a method by Lane and Eynon (*18*). In a volumetric flask, 25 mL citrus juice, 100 mL distilled water and 2 mL lead(II) acetate (45 %) (Indo Chem Laboratories, Vadodara Gujarat, India) were taken. To this, 2 mL potassium oxalate (22 %) were added and the volume was made up to 250 mL with distilled water. To 50 mL of this sample, 5 mL of concentrated HCL (Sigma-Aldrich, Merck) were added and kept for 24 h for inversion. The next day, the sample was neutralized with 40 % NaOH until it turned a light pink colour and the final volume was made up to 100 mL. In another conical flask, 2.5 mL of each Fehling's solution A and B (Sigma-Aldrich, Merck) were added and then diluted with 50 mL of distilled water and brought to the boil on a hot plate. A few drops of methylene blue indicator were added to the boiling Fehling's solution and the titration was carried out with a juice sample taken in a burette. To determine the reducing sugars, 25 mL of citrus juice were put in a 250-mL volumetric flask. Then, 2 mL of each lead(II) acetate (45 %) and potassium oxalate (22 %) (Indo Chem Laboratories) were added, the volume was made up to 250 mL by adding distilled water and the mixture was kept for 15 min to precipitate. It was then filtered in a burette. In another 250-mL conical flask, 2.5 mL of each Fehling's solution A and solution B and 50 mL of distilled water were added. Before boiling, 1–2 drops of methylene blue indicator were added and the filtrate was titrated until brick colour was obtained (*18*). Mass fractions of total, reducing and non-reducing sugars were calculated as follows:


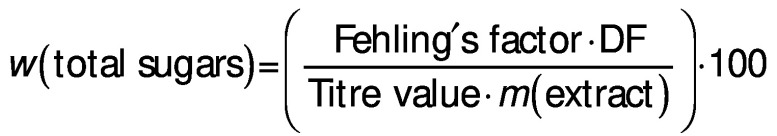
 /8/


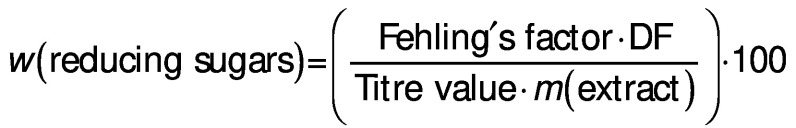
 /9/

where DF is dilution factor and Fehling’s factor (g of inverted sugar) is titre value·2.5/1000.

*w*(non-reducing sugars)=(*w*(total sugars)–*w*(reducing sugars))·0.95 /10/

### Sensory evaluation of pummelo × sweet orange hybrids

Sensory evaluation plays a pivotal role in defining product quality. The judges had the task of assessing various evaluation parameters using a nine-point hedonic scale following the guidelines established by Jellinick (*19*). These parameters included attributes such as appearance, colour, flavour, taste, consistency and overall acceptability. A semi-trained panel of ten judges, consisting of six males 25-40 years old and 4 females 25-30 years old, conducted the analysis. Initially, an organoleptic evaluation of the juice was carried out immediately after extraction. The judges documented the sensory characteristics of both the fruit and the juice on a dedicated sensory evaluation sheet. Statistical analysis was then used to decode and interpret the obtained scores so that meaningful conclusions could be drawn about the sensory attributes of the products.

### Molecular characterization of pummelo × sweet orange citrus hybrids using acidity-specific markers

Twenty-eight pummelo × sweet orange citrus hybrids and their parents were used for molecular profiling specific for acidity. Genomic DNA was isolated from leaf tissues using the cetyltrimethylammonium bromide (CTAB) method with minor modifications (*20*). A total of four gene sequences of *Citrus sinensis* specific for acidity were retrieved from the National Center for Biotechnology Information (NCBI, www.ncbi.nlm.nih.gov): sequence accession numbers MK139972.1, MK139971.1, MK139970.1 and MK139969.1 (*21*). All of the sequences were submitted to Krait, microsatellite identification and primer design, and the Krait results were carefully counted to obtain the number of repeats (*22*). The primer design parameters were as follows: primer length 20–23 bp, GC content 30–58 % and optimum primer *t*_m_=55–62 °C. Eight new expressed sequence tag-simple sequence repeat (EST-SSRs) markers were designed from the nucleotide sequences of these acidity-specific markers. These primers are located on chromosomes 3, 4 and 6 and the SSR motifs GAC and GCTT were predominant in these primers. The PCR was carried out in 12 μL of reaction mixture containing 1 μL of each primer (forward and reverse), 4 μL of 25 ng/μL genomic DNA as template, 2 μL of PCR buffer, 1 μL of deoxynucleotide triphosphates (dNTPs) mix and 0.15 μL *Taq* polymerase. The volume was made up to 12 μL with sterile distilled water. Applied Biosystems™ Veriti™ 96-well thermal cycler (Thermo Fisher Scientific) was used for thermal cycling. The PCR-amplified products were run on 3 % high-resolution agarose gels. Electrophoresis was carried out at 100 V for 3 to 4 h. The DNA profiles were visualized with a UV transilluminator and photographed with a gel documentation system (AlphaImager® Mini Gel Documentation, Radnor, PA, USA). Genetic similarity between individual pairs of genotypes was analyzed using NTSYSpc 2.1 software (*23*).

### Statistical analysis

The experimental setup followed a randomized block design (RBD) in a factorial arrangement. Each treatment consisted of three replications with one tree per replication. The collected data were subjected to statistical analysis of variance (ANOVA) using SAS v. 9.3 software (*24*). Duncan's multiple range test (DMRT) was used to compare significant differences at p≤0.05. Principal component analysis (PCA) was performed using the average concentration of the physicochemical quantitative trait to highlight the distances between genotypes, using R Studio v. 2022.07.1–554 (*25*). Correlation analysis was done for the biochemical parameters using Pearson’s correlation with the same software. The evaluation of genetic similarity among genotype pairs was conducted using the NTSYSpc 2.1 software (*23*). The average similarity across all genotype pairs served as a threshold for delineating clusters. The Dice coefficient (*26*) was used to estimate genetic similarity. Dendrograms were then generated using cluster analysis *via* the unweighted pair group method with arithmetic means (UPGMA). Phenotypic coefficient of variation (PCV) and genotypic coefficient of variation (GCV), heritability and genetic advance were estimated. The broad-sense heritability was calculated as genotypic variance/phenotypic variance (*27*).

## RESULTS AND DISCUSSION

### Juice yield, pH, TSS, TA and TSS/TA ratio

**Fig. S1:**
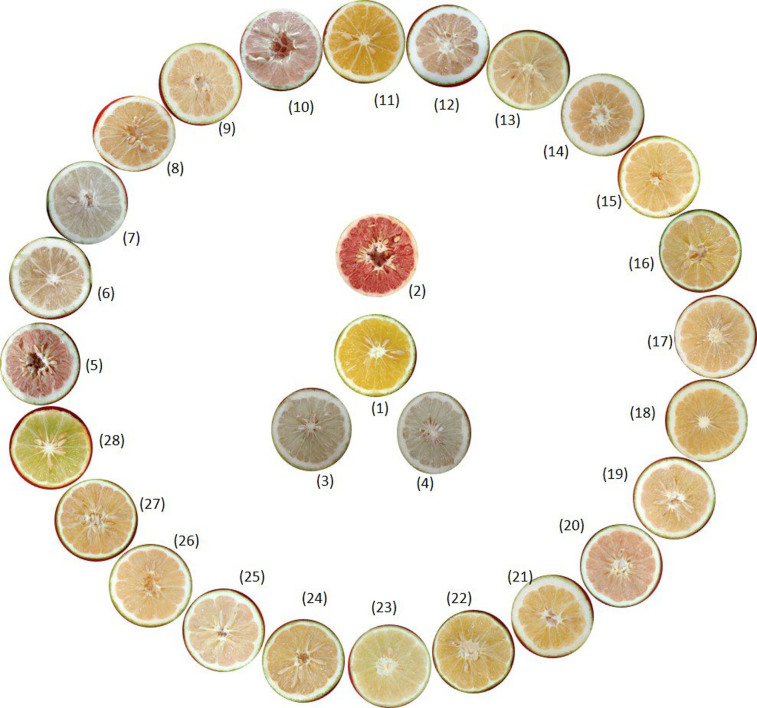
Transverse section of 24 pummelo × sweet orange hybrid fruits and their four parents. Parents:(1)=Sweet orange cv. Mosambi, (2)=PS-2 (pummelo red), (3)=PS-5 (pummelo white), (4)=PS-10 (pummelo white), hybrids: (5)=SCSH 3-10, (6)=SCSH 3-14, (7)=SCSH 3-15, (8)=SCSH 5-5, (9)=SCSH 7-12, (10)=SCSH 9-2, (11)=SCSH 9-10, (12)=SCSH 9-20, (13)=SCSH 11-15, (14)=SCSH 11-19, (15)=SCSH 13-4, (16)=SCSH 13-13, (17)=SCSH 13-14, (18)=SCSH 13-17, (19)=SCSH 15-2, (20)=SCSH 15-3, (21)=SCSH 15-18, (22)=SCSH 15-19, (23)=SCSH 17-9, (24)=SCSH 19-2, (25)=SCSH 19-6, (26)=SCSH 19-8, (27)=SCSH 21-10, (28)=CRH 20-11

Citrus juices such as pummelo, grapefruit, orange and lemon juice have distinctive flavours, aromas and nutritional profiles due to a variety of biochemical properties. The high juice volume is an important factor determining the suitability of a variety for both processing and fresh consumption (Fig. S1). The data on juice content in Table S2 show significant variations among hybrids. Among the 24 hybrids, juice volume was the highest in SCSH 5-5 (232.33 mL) and the lowest in SCSH 11-15 (56.67 mL), which is not significant compared to SCSH 9-2 (76.67 mL). Among the parents, sweet orange cv. Mosambi had the highest fruit juice content (133.33 mL), which is not significant compared to the other two parents, and the lowest in PS-10 (pummelo white; 65.67 mL).

Juice yield is an important parameter in citrus fruits. Hybrids and their parents had significantly different juice yield (Table S2). Significantly highest juice yield was obtained from SCSH13-14 (45.64 %), followed by CRH 20-11, SCSH 15-19, SCSH 11-19, SCSH 5-5 and SCSH 3-15. However, the lowest juice yield was obtained from SCSH 3-10 (25.08 %), which showed statistical parity with SCSH 17-9. Furthermore, these two parents showed statistical similarity in juice yield, while significantly higher juice yield was obtained from sweet orange cv. Mosambi than from the other three parents. Compared to their parents, hybrids exhibited intermediate values of juice yield between both female and male parents. This is well explained by Fiévet *et al*. (*28*), who states that heterosis is a result of multiple component traits, even when these traits can be entirely explained by an additive genetic model. When the component traits show phenotypic divergence in parents, the manifestation of heterosis in the complex trait is frequently observed in the progeny, even if the component traits themselves remain close to the mid-parent values. The hybrids showed significant variation in the pH of the fruit juice (Table S2). It is evident that hybrid SCSH 3-10 had the highest pH (4.00), followed by SCSH 3-14, while hybrid SCSH 15-18 (3.45) had the lowest pH, which was not significant compared to SCSH 13-13. Among the parents, Mosambi, a male parent, produced the fruit juice with the highest pH=4.73, while the juice from PS-5 (pummelo white) had the lowest pH=3.93. Different hybrids had pH values that were lower than of their parents. The data presented in Table S2 indicate that significant differences in juice total soluble solids (TSS) were found among hybrids and their parents. Significantly, the highest TSS was found in hybrid SCSH 11-15 (11.33 %), while the lowest TSS was found in hybrid SCSH 7-12 (7.33 %), which was not significantly different from SCSH 19-8, SCSH 9-20, SCSH 15-2, SCSH 15-3, SCSH 15-18, SCSH 15-19, SCSH 17-9 and SCSH 21-10. Among the parents, the highest TSS (11.00 %) was found in Mosambi, the male parent.

The acid content (titratable acidity (TA)) is another important characteristic that affects the juice quality of citrus fruits. The highest TA was measured in SCSH 11-15 (2.11 %) and the lowest in SCSH 7-12 (1.00 %). In addition, three parents showed statistical similarity in TA, while PS-2 (pummelo red) had significantly higher TA (1.50 %) than all other parents. Compared to the parents, the hybrids showed both higher and lower TA values (Table S2). The TA of the citrus juices was essentially in an inverse relationship with the corresponding pH values (*29*). The content of TSS and TA as well as the fine balance between them is of great importance for defining the flavour and intrinsic quality of citrus fruits. In the present study, SCSH 13-14 (9.57) had the highest TSS/TA ratio, followed by SCSH 13-17, while it was the lowest in SCSH 21-10 hybrid (4.09). Among parents, two parents showed statistical similarity in TSS/TA, while the female parent PS-10 (pummelo white) was found to have significantly higher ratio than the other three parents. Paithankar *et al.* (*30*) reported TSS value of 8.39 %, acidity of 7.18 %, mass of 54.31 g, juice content of 52.41 % and ascorbic acid mass concentration of 31.47 mg/100 g in Sarbati lime.

### Ascorbic acid, total flavonoid and total phenolic content, total antioxidant activity and sugar mass fractions

The ascorbic acid concentration in the fruit juice of citrus hybrids and their parents showed clear differences (Table S2). The highest concentration of ascorbic acid was observed in SCSH 17-9 (58.13 mg/100 g), followed by SCSH 11-15, SCSH 15-18, SCSH 11-15 and CRH 20-11, while hybrid SCSH 11-19 (40.00 mg/100 g) had the lowest ascorbic acid concentration. Moreover, among the parents, significantly highest ascorbic acid concentration was found in the fruit juice of sweet orange cultivar Mosambi, a male parent, while all three female parents had lower values than Mosambi, but without significant differences from each other. Furthermore, none of the hybrids had higher values of ascorbic acid than either of the parents, showing intermediate values of ascorbic acid concentration.

Total phenolic content (TPC) expressed as GAE in fruit juice showed notable differences between the hybrids and their parents (Table S2). The fruit juice of the hybrid SCSH 13-13 had significantly highest TPC expressed as GAE (107.33 mg/100 g). On the other hand, it was the lowest in SCSH 3-10 (40.67 mg/100 g), which showed statistical parity with SCSH 5-5 and SCSH 3-15. Among the parents, the highest TPC (168.17 mg/100 g) was found in PS-2 (pummelo red), while the lowest (96.83 mg/100 g) was found in PS-10 (pummelo white).

Data in Table S2 related to the total flavonoid content (TFC), expressed as QE, in fruit juice showed significantly highest value in hybrid SCSH 11-15 (59.33 mg/100 g), followed by SCSH 11-19 and SCSH 3-15, while the lowest TFC was found in hybrid SCSH 15-18 (23.67 mg/100 g), which did not differ significantly from SCSH 5-5, SCSH 15-19, SCSH 7-12, SCSH 19-6, SCSH 13-4, SCSH 13-13, SCSH 13-14 and SCSH 13-17. Among the parents, the highest TFC was found in PS-10 (pummelo white), a female parent.

Total antioxidant activity of the fruit juice of hybrids is shown in Table S2. It varied significantly among the hybrids, with SCSH 9-2 showing the highest DPPH radical scavenging mean value, expressed as Trolox equivalents, of 5.62 µmol/g followed by SCSH 13-14, SCSH 3-15 and SCSH 3-14. On the other hand, CRH 20-11 was found to have the lowest DPPH radical scavenging mean value of 2.03 µmol/g. In contrast, PS-2 (pummelo red) had the highest total antioxidant activity (4.14 µmol/g) among the parent plants, while Mosambi had the lowest total antioxidant activity of 1.83 µmol/g.

The data on sugar mass fraction clearly indicated substantial differences between the hybrids and their parent varieties (Table S2). Significantly highest total sugar mass fraction was measured in the fruit juice of hybrid SCSH 13-14 (5.76 %), while hybrid SCSH 11-15 had the lowest value (2.05 %). Among the parents, the highest total sugar mass fraction was found in PS-5 (pummelo white) and the lowest in PS-2 (pummelo red). The highest reducing sugar mass fraction was in hybrid CRH 20-11 (2.78 %) and the lowest in SCSH 11-15 and SCSH 15-2 (1.69 %), followed by SCSH 3-10. Among the parents, male cultivar had higher reducing sugar content (2.98 %) than the female parents. Non-reducing sugar mass fraction was also calculated and it was the highest in SCSH 13-14 (3.17 %) and the lowest in SCSH 11-15. The hybrids showed intermediate reducing values compared to the parents. Rehman *et al*. (*29*) studied nutritional properties of wild citrus species and reported the following results of phytochemical analysis: total phenolic content, as GAE, 132–243 mg/100 g, total flavonoid content, as QE,4.2–12.1 mg/100 g, vitamin C 36.3–62.3 mg/100 g, pH=2.9–5.8, total soluble solids 8.1–13.7 %, titratable acidity 7.9–13.1 % and total sugar content 5.1–8.8 %.

### Sensory acceptability of the pummelo × sweet orange hybrids

The decisive criterion for the desirability of a food product for the consumer is its flavour. According to Sharma *et al*. (*31*), quality is as a crucial parameter in evaluating the suitability of any food product for human consumption. The quality of the product can be determined using standard methods in terms of sensory quality, shelf life and microbial growth. According to Thakkar and Shah (*32*), sensory analysis of food is becoming increasingly important in evaluating the acceptability of a particular food. Sensory analysis is a technique that utilizes human testers as a measurement tool. A numerical scoring test is used to analyze the characteristics of one or more samples (*33*). The sensory property serves as an important parameter in assessing the quality of a product. Currently, sensory evaluation has become an indispensable tool in the food industry and plays a crucial role in interacting with the key sectors of food production (*34*).

The surface characteristics of a food item contribute to its appearance. The mean score for the appearance was measured, with the highest mean score of 9.0 for hybrids SCSH 15-3, CRH 20-11 and Mosambi, and the lowest mean score of 6.3 for parent PS-10 (pummelo white) (Table 1). Colour is a very important attribute for the evaluation of the overall quality of fruit and juice. The mean scores for the fruit colour ranged from 6.3 to 9.0. Hybrid SCSH 9-20 and parent PS-2 (pummelo red) had the highest mean score (9.0), while the hybrid SCSH 13-17 and parent PS-10 (pummelo white) obtained 6.3.

**Table 1:** Mean scores obtained for sensory analysis of fresh fruits

Hybrid or parent	Appearance	Colour	Flavour	Texture	Taste	Overall acceptability
SCSH 3-10	8.7	8.7	9.0	8.3	9.0	9.0
SCSH 3-14	7.0	6.7	7.7	7.7	7.0	7.3
SCSH 3-15	8.3	8.3	8.7	7.0	7.7	8.0
SCSH 5-5	6.7	7.7	7.7	7.3	8.3	8.0
SCSH 7-12	7.3	7.3	8.0	8.3	7.7	8.3
SCSH 9-2	8.3	8.7	8.7	8.3	8.0	8.7
SCSH 9-10	6.3	8.3	7.7	7.3	7.0	6.7
SCSH 9-20	8.3	9.0	8.7	8.3	8.7	8.7
SCSH 11-15	7.3	8.3	8.3	8.0	7.7	7.7
SCSH 11-19	7.7	7.7	7.7	8.0	7.3	7.7
SCSH 13-4	8.3	8.3	8.3	7.7	7.3	8.0
SCSH 13-13	7.7	6.7	7.0	6.7	5.3	6.7
SCSH 13-14	8.3	8.3	8.3	7.7	7.0	7.3
SCSH 13-17	6.7	6.3	7.3	7.0	6.3	6.7
SCSH 15-2	8.7	8.3	8.3	8.7	8.0	8.7
SCSH 15-3	9.0	8.7	8.7	8.0	9.0	8.7
SCSH 15-18	8.3	8.3	8.3	8.3	8.3	8.3
SCSH 15-19	8.0	8.3	7.7	7.3	7.0	6.7
SCSH 17-9	7.7	7.7	7.3	7.0	6.3	7.3
SCSH 19-2	8.3	8.3	8.0	8.3	8.7	8.3
SCSH 19-6	8.3	7.7	7.3	7.0	8.0	7.3
SCSH 19-8	7.7	6.7	7.3	7.7	7.7	7.7
SCSH 21-10	7.7	7.7	7.7	7.3	7.3	7.3
CRH 20-11	9.0	8.7	8.3	8.7	9.0	8.7
Parentage						
Sweet orange cv. Mosambi	9.0	8.3	9.0	8.3	9.0	9.0
PS-2 (pummelo red)	8.7	9.0	9.0	8.3	8.7	8.7
PS-5 (pummelo white)	6.7	6.7	7.0	7.7	6.3	6.7
PS-10 (pummelo white)	6.3	6.3	6.7	7.7	6.0	6.3
Mean	7.9	7.9	8.0	7.8	7.6	7.8
						

According to Rowe (*35*), flavour is one of the central attributes that imparts identifiable character to the food item. Regarding the flavour of the hybrids, the mean score ranged from 6.7 to 9.0. Hybrid SCSH 3-10, male parent Mosambi and female parent PS-2 (pummelo red) had the highest mean score (9.0), followed by SCSH 3-10, SCSH 9-2, SCSH 9-20 and SCSH 15-3, and the lowest score was obtained for PS-10 (pummelo white) female parent. Texture is a physical property perceived by the eyes and the skin and muscle senses in the mouth. Hybrids SCSH 15-2 and CRH 20-11 had the highest mean score of 8.7 for the texture. The lowest mean score (6.7) was obtained for SCSH 13-13. Taste is an aesthetic appreciation in the mouth and it is the most important sensory characteristic that determines the acceptability of fruit and juice. The selected pummelo × sweet orange hybrids were tested by a sensory panel, who gave the hybrids SCSH 3-10, SCSH 15-3, CRH 20-11 and male parent Mosambi the highest taste mean score of 9.0, followed by SCSH 19-2 and PS-2 (pummelo red) with the mean scores of 8.7 (Table 1). The overall acceptability of the fruits was analyzed by sensory analysis and maximum mean score was given to SCSH 3-10 and Mosambi, followed by SCSH 9-2, SCSH 9-20, SCSH 15-2, SCSH 15-3, CRH 20-11 and PS-2 (pummelo red) parent with the mean score of 8.7. PS-10 (pummelo white) had the lowest mean score (6.3). Previously, similar sensory evaluation studies were done by Lego *et al*. (*36*) in Sikkim mandarin and by Idangodage *et al*. (*37*) in *Citrus madurensis*.

### Heritability studies

In this study, a broad spectrum of variability was observed in all fruit juice properties. The observed phenotypic coefficient of variation (PCV) for all 13 properties exceeded the respective genotypic coefficient of variation (GCV), suggesting that in addition to genetic factors, environmental factors also contribute significantly to the observed variations. This is consistent with previous results reported by Mishra *et al*. (*38*). Moreover, the small difference between these two estimates (GCV and PCV) indicates a lower impact of the environment on the observed variability. The highest PCV was observed for non-reducing sugar (44.47 %), followed by juice volume (36.91 %), total antioxidant activity (33.22 %), total phenolic content (31.83 %) and total flavonoid content, while TSS/TA, juice yield, TA, total sugars, reducing sugars, ascorbic acid, TSS and pH showed a low PCV (25.56, 20.80, 24.49, 22.73,19.73, 16.08, 13.72 and 7.01 %, respectively). GCV was estimated to be high (*>*30 %) for non-reducing sugars, juice volume, total antioxidants and total phenolic content, moderate (20–30 %) for total flavonoids, TSS/TA and juice yield, while it was low (*<*20 %) for total sugars, TA, reducing sugars, TSS, ascorbic acid and juice pH (Table 2). Citrus species show a high degree of heterozygosity, resulting in extensive genotypic and phenotypic diversity in their first-generation progeny (*39*, *40*). The current study revealed a higher heritability (≥80 %) of all juice properties, which strengthens the evidence of limited impact of environmental factors on these traits. The high values of both GCV and heritability (>80 %) suggest the potential for successful selection to improve these traits. In addition, a high genetic advance (GA) as a percentage of the mean was estimated for non-reducing sugars, juice volume, total phenolic content and total antioxidants, further indicating the prospects for improvement through selection for these specific traits (Table 2). Juice properties such as pH, total sugars, reducing sugars, non-reducing sugars and total phenolic content show high heritability and genetic advance as a percentage of the mean, suggesting that these attributes are primarily determined by additive genes with less influence from the environment. This suggest that they are suitable for the selection of hybrid genotypes based on these traits (*41*). On the other hand, non-additive gene action could be responsible for total flavonoid, total sugar, reducing sugar, ascorbic acid and TSS contents, TSS/TA, juice yield, titratable acidity and juice pH, as shown by low to moderate GA (20–30 %) along with high heritability (*>*80 %), which could be improved in fruit juice by heterosis breeding as reported for nutrient content in banana (*27*). Previously, Devarajan *et al.* (*27*) and Karmakar *et al*. (*42*) reported the role of both additive and non-additive gene action for mineral content in banana pulp and ridge gourd, respectively. The outcomes of that study affirm a stronger impact of genetic factors on the properties of citrus fruit juice. Numerous recent experimental studies have investigated GCV, PCV and heritability in vegetative traits for grapefruit genotypes (*43*) and mandarin (*44*). Additionally, studies on disease resistance traits in citrus hybrids (*45*) and physico-biochemical fruit quality traits in different *Citrus* species have been conducted (*44*, *46*, *47*). At the biochemical level, complex phenotypes result from the interaction of multiple metabolites over time (*28*). The concentrations of metabolites are themselves a complex phenotype as they are influenced by the enzyme quantities and activities within metabolic pathways, along with the flux, which indicates the rate of turnover through the pathway (*28*, *48*-*50*). To the best of our knowledge, this is the first study on interspecific hybrids of citrus (pummelo × sweet orange) focused on the estimation of heritability of juice properties.

**Table 2:** Estimation of descriptive statistics, heritability and genetic advance of the pummelo × sweet orange juice properties

Juice property	Range		Mean	SEM	CV/%	GCV/%	PCV/%	H^2^/%	GA	GAM/%
*V*(juice)/mL	50–280		126.7	8.73	24.75	34.91	36.89	0.89	86.22	68.05
Recovery/%	15.11–51.36		35.02	2.18	6.2	21.97	24.49	0.8	14.22	40.61
TSS/%	6–12		8.72	0.33	0.95	10.87	12.75	0.72	1.66	19.08
pH	3.4–4.7		3.77	0.02	0.07	6.89	7.01	0.96	0.52	13.97
TA/%	0.81–2.36		1.52	0.11	0.33	18.41	22.73	0.65	0.46	30.71
TSS/TA	3.3–10.85		6.01	0.44	1.25	22.14	25.56	0.75	2.37	39.53
*w*(ascorbic acid)/(mg/100 g)	36.80–66.40		47	2.33	6.61	10.7	13.72	0.6	8.08	17.20
TPC as *w*(GAE)/(mg/100 g)	37–181		79.44	4.38	12.41	30.37	31.83	0.91	47.41	59.68
TFC as *w*(QE)/(mg/100 g)	19–73		38	4.44	12.6	24.26	31.61	0.58	14.58	38.37
DPPH as *b*(Trolox)/(µmol/g)	1.53–7.34		3.56	0.24	0.68	31.05	33.22	0.87	2.13	59.79
*w*(total sugar)/%	2.08–6.00		3.98	0.09	0.25	19.3	19.71	0.95	1.55	38.96
*w*(reducing sugar)/%	1.29–3.20		2.19	0.05	0.15	15.46	16.08	0.92	0.67	30.64
*w*(non-reducing sugar)/%	0.40–3.57		1.7	0.09	0.27	43.49	44.47	0.95	1.57	87.63

SEM=standard error of the mean, CV=coefficient of variation, GCV=genotypic coefficient of variation, PCV=phenotypic coefficient of variation, H^2^=broad sense heritability, GA=genetic advance, GAM=mean value of genetic advance, JR=juice recovery, TSS=total soluble solid, TA=titratable acidity, TPC=total phenolic content, GAE=gallic acid equivalent, TFC=total flavonoid content, QE=quercetin equivalent

### Correlation, PCA and cluster analysis

**Fig. 1:**
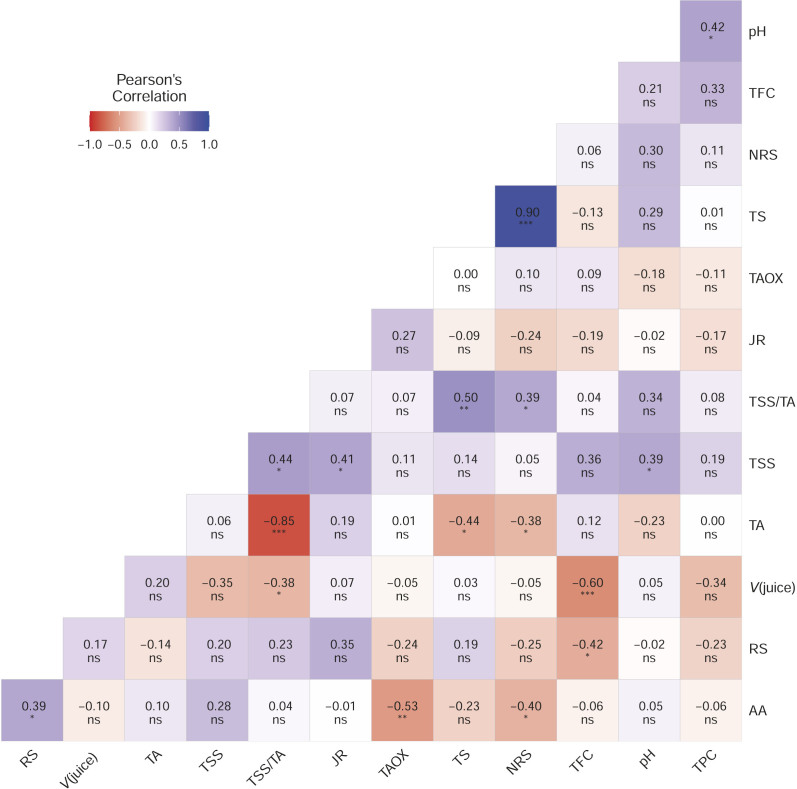
Pearson’s correlation coefficients for pummelo × sweet orange juice properties. ns=not significant (p≥0.05), *p<0.05, **p<0.01 and ***p<0.001

Pearson’s correlation coefficients among the different juice properties are shown in Fig. 1. The pH had positive correlation with TPC (r=0.42, p<0.05), total sugars (TS) correlated positively with non-reducing sugars (NRS) (r=-0.90, p<0.001),TSS/TA ratio correlated positively with TS (r=-0.50, p<0.01) and NRS (r=-0.39, p<0.05) but negatively with titratable acidity (TA) (r=-0.85, p<0.001), total soluble solids (TSS) showed positive correlation with pH, juice recovery (JR), TSS/TA (r=0.39, p<0.05; r=0.41, p<0.05; r=0.44, p<0.05), TA had negative correlation with TS (r=0.44, p<0.05) and NRS (r=0.38, p<0.05), juice volume (*V*_juice_) strongly negatively correlated with total flavonoid content (TFC) (r=0.60, p<0.001), reducing sugars (RS) negatively correlated with TFC (r=0.42, p<0.05), ascorbic acid (AA) content positively correlated with RS (r=0.39, p<0.05) and showed linearly negative correlation with NRS (r=0.40, p<0.05) and total antioxidants (DPPH) (r=0.53, p<0.01) (Fig. 1).

**Fig. 2:**
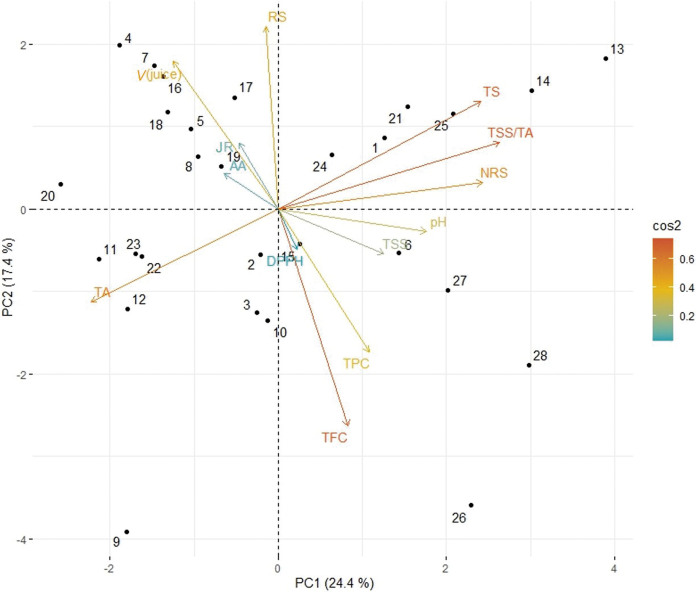
PCA-biplot showing different genotypes:1=SCSH 3-10 2=SCSH 3-14, 3=SCSH 3-15, 4=SCSH 5-5, 5=SCSH 7-12, 6=SCSH 9-2, 7=SCSH 9-10, 8=SCSH 9-20, 9=SCSH 11-15, 10=SCSH 11-19, 11=SCSH 13-4, 12=SCSH 13-13, 13=SCSH 13-14, 14=SCSH 13-17, 15=SCSH15-2, 16=SCSH 15-3, 17=SCSH 15-18, 18=SCSH 15-19, 19=SCSH 17-9, 20=SCSH 19-2, 21=SCSH 19-6, 22=SCSH 19-8, 23=SCSH 21-10, 24=CRH 20-11, 25=sweet orange cv. Mosambi, 26= PS-2 (pummelo red), 27=PS-5 (pummelo white), 28=PS-10 (pummelo white), and juice properties: *V*(juice), juice recovery (JR), total soluble solids (TSS), pH, titratable acidity (TA), TSS/TA, ascorbic acid (AA), total phenolic content (TPC), total flavonoid content (TFC), total antioxidants (DPPH), total sugars (TS), reducing sugar (RS) and non-reducing sugars (NRS) along the two major principal components (PC1 and PC2)

PCA was performed using the mean values of different properties of the juice of 24 citrus hybrids and four parental genotypes. Considering a minimum threshold eigenvalue of one, the five PCs (*V*_juice_, JR, TSS, juice pH and TA) were considered, which explained 71.16 % of the total variation between the analyzed citrus hybrids and their parents (Fig. 2). PC1 contributed 24.43 % of the total variation, followed by PC2 (17.40 %). Similarly, these parameters were found to be important in the study by Dubey *et al*. (*51*). Cluster analysis clearly showed that cluster A1 consists mainly of hybrids that have good juice volume. However, hybrids SCSH 13-15 and SCSH 11-15, which perform excellently, were presented as an outgroup (Table 3).

**Table 3:** Mean values of the juice properties of the clusters of citrus fruit hybrids and their parents obtained on the basis of their biochemical traits

Juice parameter	*N*(group)_mean_				*N*(population)_mean_
Cluster A		Cluster B	
A1	A2	B1	B2	
*V*(juice)/mL	205.93	128.45	56.67	94.50	121.39
Recovery/%	36.78	34.33	42.39	35.00	37.12
TSS/%	8.27	8.42	11.33	9.10	9.28
pH	3.78	3.67	3.66	3.89	3.75
TA/%	1.60	1.56	2.11	1.41	1.67
TSS/TA	5.32	5.72	5.38	6.73	5.79
*w*(ascorbic acid)/(mg/100 g)	44.69	47.70	54.67	45.52	48.14
TPC as *w*(GAE)/(mg/100 g)	60.88	71.83	85.00	99.37	79.27
TFC as *w*(QE)/(mg/100 g)	28.53	35.79	59.33	43.08	41.68
DPPH as *b*(Trolox)/(µmol/g)	3.76	3.53	4.43	3.58	3.83
*w*(total sugar)/%	4.11	3.86	2.10	4.28	3.59
*w*(reducing sugar)/%	1.78	2.21	1.69	2.19	1.97
*w*(non-reducing sugar)/%	1.78	1.57	0.39	1.99	1.43

TSS=total soluble solid, TA=titratable acidity, TPC=total phenolic content, GAE=gallic acid equivalent, TFC=total flavonoid content, QE=quercetin equivalent

**Fig. 3:**
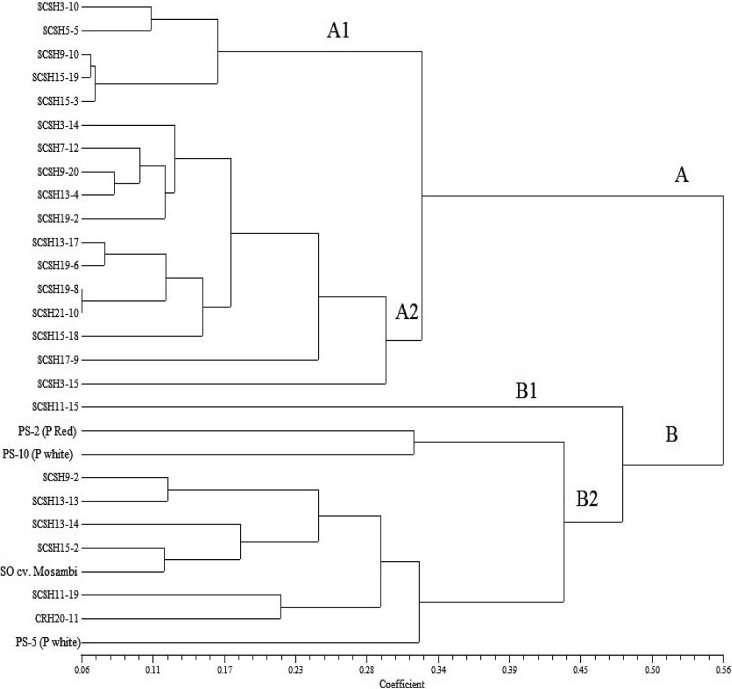
Dendrogram showing relationships between the 24 citrus fruit hybrids (SCSH) and their parents (PS)

A dendrogram was created from the pairwise distance matrices to determine the parameter relationships and percentage similarities (Fig. 3). All 24 interspecific hybrids along with their parents were grouped into two main clusters A and B, with a similarity value of 0.56 %. Cluster A comprised most of the studied genotypes and was further divided into two clusters A1 and A2 with a similarity value of 60.71 % and cluster B comprised the remaining genotypes and further divided into two clusters B1 and B2 with a similarity value of 39.29 %. Citrus hybrids SCSH 3-15 and SCSH 11-15 were classified as an outgroup based on biochemical traits. Grouping by biochemical parameters showed that most of the hybrids were closely related to the female parent (pummelo red). In the present study, some of the pummelo × sweet orange hybrids proved to be superior to their parental genotypes in the contents of antioxidants, vitamin C, phenolics and flavonoids in the juice. The heterotic effect for nutrients in edible tissues has previously been reported in several food crops (*42*, *52*, *53*). Nevertheless, antioxidants, vitamin C and other nutrients were found to be intermediate in several hybrids compared to their parents. This phenomenon is attributed to non-transgressive segregation, where alleles at multiple loci from different parental populations recombine in hybrids, resulting in intermediate trait expression (*54*). Cluster analysis effectively categorized citrus genotypes into different groups based on their juice nutrient concentrations. Interestingly, the results indicated that the identification of genotype groups belonging to the same genomic or parentage group was not possible, suggesting that hybrids showed a mixture of characteristics related to their juice mineral content. These findings are consistent with previous studies on minerals and fruit quality traits observed in banana (*27*), citrus (*55*) and apricot (*56*).

### Molecular characterization using newly generated acidity specific markers

**Fig S2:**
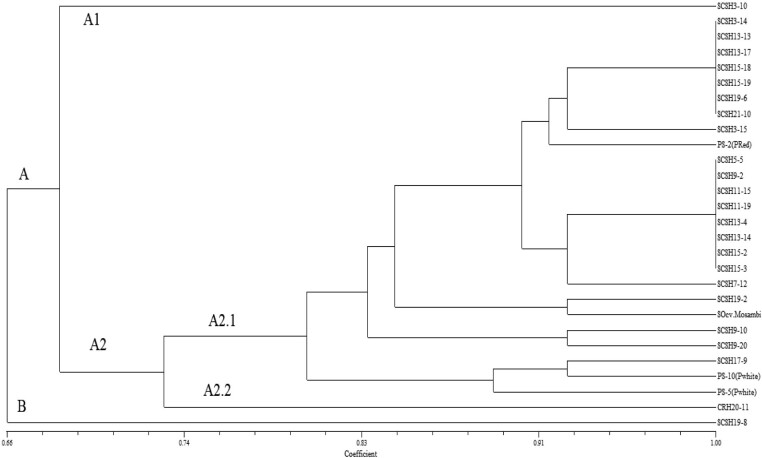
Genetic tree based on acidity-specific markers of 24 citrus fruit hybrids and their four parents

The use of conventional breeding methods for citrus fruits is limited by the long generation time, usually from 3 to 7 years. When breeding for quality traits such as fruit acidity, the evaluation period is longer. Therefore, the identification of trait-linked markers is of great benefit to citrus breeders. A total of eight primers were designed and synthesized for molecular profiling of important citrus genotypes specific for acidity. Out of eight primers, seven primers, namely NRCH2, NRCH3, NRCH4, NRCH5, NRCH6, NRCH7 and NRCH8 were polymorphic and showed a total polymorphism of 87.5 % and primer NRCH1 was not amplified (Table S3). The amplicon size ranged from 100 to 190 bp. In a detailed study based on molecular data, all 28 citrus genotypes were grouped into one major cluster A (96.43 %) and one outgroup B (3.57 %) on the basis of acidity-specific primers (Fig. S2). Cluster A comprised 23 hybrids and all four parents. Cluster A was further subdivided into one outgroup A1, which included only one hybrid NRCH 3-10, and A2, which included most hybrids and all parents. In outgroup B, only hybrid SCSH 19-8 was present. The sweet orange is originally a natural hybrid of mandarin and pummelo. Most cultivars are the result of somatic mutation from one ancestor tree. The acidity values of the progeny of all three cross combinations [PS-2 (pummelo red) × sweet orange cv. Mosambi, PS-5 (pummelo white) × sweet orange cv. Mosambi, PS-10 (pummelo white) × sweet orange cv. Mosambi] are listed in Table S4. The variability of acidity is high; 42.85 % hybrids (SCSH 3-14, SCSH 3-15, SCSH 5-5, SCSH 9-20, SCSH 11-15, SCSH 11-19, SCSH 13-4, SCSH 13-13, SCSH 15-19, SCSH 19-2, SCSH 19-8 and SCSH 21-10) had high acidity (>1.50 %), and medium acidity (1.00–1.50 %) was found in 57.15 % genotypes (SCSH 3-10, SCSH 7-12, SCSH 9-2, SCSH 9-10, SCSH 13-14, SCSH 13-17, SCSH 15-2, SCSH 15-3, SCSH 15-18, SCSH 17-9, SCSH 19-6, CRH 20-11 and all four parents). In the present study, a substantial variation in acidity status was observed. Molecular characterization of pummelo × sweet orange hybrids showed that >50 % of the hybrids were grouped with the parents with medium acidity (*57*). Sometimes there is complementation of genes and alleles (from different parents), resulting in the chance that a hybrid is better than the parents. There is also a phenomenon of over dominance, where F1 hybrid becomes better than the parents. There is also the possibility of additive effect (accumulation of different quantitative trait loci (QTLs)) of genes/QTLs in hybrids for quantitative traits (*58*).

## CONCLUSIONS

The results of this study show that the new developed orangelo hybrids are a valuable source of antioxidants that may fulfil additional nutritional requirements for a healthy body. Moreover, the study showed significant differences in nutritional value in both the parental varieties and the resulting hybrid genotypes. The overall acceptability of the fruits was found in SCSH 3-10 and Mosambi followed by SCSH 9-2, SCSH 9-20, SCSH 15-2, SCSH 15-3, CRH 20-11 and PS-2 (pummelo red) parent. In the present study, a higher heritability (≥80 %), which was also estimated for all juice properties, confirms the weak influence of environmental factors. On the basis of titratable acidity, juice yield, and total soluble solid and vitamin C contents, citrus hybrid SCSH 11-15 proved to be superior and hybrid SCSH 3-15 the best in terms of total antioxidant and total flavonoid contents.

## Appendix

**Table S1:** The pummelo × sweet orange hybrids and parents evaluated in the present study

Hybrid	Parent	Planting year
SCSH 3-10	Red pummelo × sweet orange cv. Mosambi	2016
SCSH 3-14		2015
SCSH 3-15		2016
SCSH 5-5		2016
SCSH 9-2		2012
SCSH 9-20		2016
SCSH 13-13		2016
CRH 20-11		2015
SCSH 13-14		2016
SCSH 11-15	White pummelo × sweet orange cv. Mosambi	2013
SCSH 11-19		2012
SCSH 13-4		2013
SCSH 7-12		2013
SCSH 9-10		2012
SCSH 13-17		2012
SCSH 15-2		2012
SCSH 15-3		2014
SCSH 15-18		2014
SCSH 15-19		2012
SCSH 17-9		2014
SCSH 19-2		2012
SCSH 19-6		2014
SCSH 19-8		2014
SCSH 21-10		2012
Parentage		
Sweet orange cv. Mosambi	♂	2003
PS-2 (pummelo red)	♀	2003
PS-5 (pummelo white)	♀	2003
PS-10 (pummelo white)	♀	2003

**Table S2:** The physicochemical traits of citrus fruit hybrids (pummelo × sweet orange) and their parents

Hybrid	*V*(juice)/ mL	JR/ %	pH	TSS/%	TA/ %	TSS/TA	w(AA)/ (mg/100 g)	TPC as *w*( GAE)/(mg/100 g)	TFC as *w*( QE)/(mg/100 g)	DPPH as *b*( Trolox)/(µmol /g)	*w*(TS)/%	*w*(RS)/%	*w*(NRS)/%
SCSH 3-10	(199.7±10.8)^b^	(25.1±2.3)^kl^	(4.00±0.03)^d^	(8.7±0.6)^cde^	(1.37±0.07)^ghijk^	(6.5±0.6)^defg^	(45.9±3.2)^defghi^	(40.8±3.2)^m^	(38.0±3.7)^efghij^	(3.9±0.2)^bcd^	(4.82±0.02)^c^	(1.75±0.03)^k^	(2.92±0.05)^ab^
SCSH 3-14	(153.0±1.7)^c^	(39.8±1.2)^bcdef^	(3.94±0.02)^d^	(9.0±0.0)^cd^	(1.6±0.3)^cdefgh^	(5.9±0.4)^efghij^	(43.2±0.8)^hi^	(70.0±3.1)^hij^	(45.3±3.4)^bcdefg^	(5.4±0.1)^a^	(3.60±0.04)^gh^	(2.11±0.07)^hi^	(1.42±0.09)^i^
SCSH 3-15	(101.7±2.9)^fgh^	(41.7±2.0)^abc^	(3.72±0.02)^ghij^	(9.0±0.0)^cd^	(1.7±0.2)^bcdef^	(5.3±0.4)^ghijkl^	(42.3±2.2)^hi^	(51.0±1.8)^lm^	(52.0±4.9)^abcd^	(5.5±0.3)^a^	(3.89±0.04)^f^	(1.96±0.01)^i^	(1.84±0.06)^g^
SCSH 5-5	(232±14)^a^	(42.7±3.5)^abc^	(3.83±0.01)^e^	(8.3±0.6)^de^	(1.79±0.02)^abcde^	(4.7±0.4)^jkl^	(42.0±1.2)^hi^	(48.2±3.8)^m^	(24.0±2.0)^kl^	(3.6±0.4)^cd^	(3.94±0.08)^f^	(2.31±0.02)^efg^	(1.55±0.09)^hi^
SCSH 7-12	(130.0±5.0)^cde^	(40.0±2.8)^bcde^	(3.57±0.04)^kl^	(7.3±0.5)^f^	(1.0±0.2)^l^	(7.4±0.4)^cd^	(42.7±1.0)^hi^	(64.5±4.8)^ij^	(28.3±2.6)^kl^	(4.5±0.7)^b^	(2.72±0.04)^l^	(2.2±0.1)^fgh^	(0.48±0.03)^l^
SCSH 9-2	(76.7±2.9)^ijk^	(33.7±2.2)^fghi^	(3.76±0.07)^efgh^	(9.3±0.6)^bc^	(1.35±0.07)^ghijk^	(6.9±0.1)^cdef^	(40.8±0.0)^hi^	(87.0±7.2)^de^	(38.0±2.2)^efghij^	(5.6±0.2)^a^	(4.2±0.1)^de^	(2.22±0.03)^fgh^	(1.9±0.2)^g^
SCSH 9-10	(198.3±18.9)^b^	(37.7±3.2)^cdefg^	(3.73±0.04)^fghi^	(8.3±0.6)^de^	(1.4±0.1)^fghij^	(5.8±0.5)^fghij^	(43.5±4.1)^ghi^	(63.7±5.7)^ijk^	(26.3±1.8)^jkl^	(4.4±0.1)^b^	(3.5±0.1)^hi^	(2.56±0.02)^c^	(0.89±0.04)^k^
SCSH 9-20	(130.0±10.0)^cde^	(29.1±3.2)^hijk^	(3.8±0.1)^ef^	(8.0±0.0)^ef^	(1.64±0.03)^bcdefg^	(4.9±0.1)^hijkl^	(50.1±4.7)^bcdef^	(70.3±4.3)^ghij^	(30.3±2.1)^hijkl^	(3.6±0.1)^cd^	(4.0±0.1)^ef^	(2.4±0.2)^de^	(1.5±0.1)^hi^
SCSH 11-15	(56.7±4.6)^k^	(42.4±3.8)^abc^	(3.7±0.2)^ij^	(11.3±0.6)^a^	(2.1±0.1)^a^	(5.4±0.5)^ghijk^	(54.7±0.9)^ab^	(85.0±6.8)^def^	(59.3±5.8)^a^	(4.4±0.2)^b^	(2.10±0.01)^m^	(1.7±0.0)^k^	(0.4±0.0)^l^
SCSH 11-19	(83.3±7.6)^hij^	(42.7±3.8)^abc^	(3.54±0.05)^l^	(9.0±0.0)^cd^	(1.83±0.04)^abcd^	(4.9±0.1)^hijkl^	(40.0±3.2)^i^	(79.7±6.5)^efgh^	(54.7±5.6)^abc^	(4.3±0.1)^b^	(4.34±0.05)^d^	(2.30±0.01)^efg^	(1.9±0.1)^fg^
SCSH 13-4	(151.7±14.9)^c^	(37.3±2.4)^cdefg^	(3.68±0.04)^hij^	(8.0±0.0)^ef^	(1.9±0.4)^ab^	(4.23±1.04)^kl^	(45.87±2.01)^defghi^	(82.9±5.3)^ef^	(35.0±2.6)^fghijkl^	(2.8±0.1)^fghi^	(3.4±0.2)^hij^	(1.95±0.05)^ij^	(1.4±0.1)^ij^
SCSH 13-13	(93.3±8.4)^fghi^	(33.1±1.2)^ghi^	(3.44±0.04)^m^	(8.0±0.0)^ef^	(1.8±0.5)^abcd^	(4.6±0.1)^jkl^	(43.5±2.4)^ghi^	(107.3±9.7)^bc^	(34.0±3.4)^fghijkl^	(4.4±0.4)^b^	(3.23±0.02)^jk^	(2.20±0.03)^gh^	(0.98±0.01)^k^
SCSH 13-14	(86.0±8.5)^hij^	(45.6±2.9)^ab^	(3.76±0.03)^efgh^	(10.0±0.0)^b^	(1.0±0.2)^kl^	(9.6±0.7)^a^	(47.3±2.3)^cdefgh^	(69.6±6.4)^hij^	(33.3±3.3)^fghijkl^	(5.6±0.5)^a^	(5.8±0.2)^a^	(2.42±0.03)^cde^	(3.2±0.2)^a^
SCSH 13-17	(111.7±2.9)^efg^	(34.2±0.6)^efghi^	(3.75±0.03)^fgh^	(10.0±0.0)^b^	(1.16±0.07)^jkl^	(8.6±0.5)ab	(44.8±3.5)^defghi^	(74.3±3.4) ^f ghi^	(33.7±3.2)^fghijkl^	(3.3±0.2)^defg^	(5.3±0.2)^b^	(2.4±0.1)^def^	(2.8±0.1)^b^
SCSH 15-2	(90.0±8.2)^ghij^	(40.5±6.5)^bcd^	(3.76±0.01)^efgh^	(8.0±0.0)^ef^	(1.33±0.03)^ghijkl^	(6.0±0.6)^efghi^	(44.3±1.7)^efghi^	(81.4±5.0)^efgh^	(33.3±3.3)^fghijkl^	(2.8±0.2)^fghi^	(3.78±0.1)^fg^	(1.69±0.01)^k^	(1.9±0.1)^fg^
SCSH 15-3	(198.3±19.7)^b^	(34.5±3.3)^defgh^	(3.5±0.0)^l^	(8.0±0.0)^ef^	(1.5±0.2)^efghij^	(5.6±0.8)^ghij^	(50.0±3.6)^bcdefg^	(75.9±6.6)^efghi^	(30.0±1.7)^ijkl^	(3.5±0.1)^cde^	(3.89±0.01)^f^	(2.5±0.1)^cd^	(1.3±0.1)^ij^
SCSH 15-18	(135.0±5.0)^cde^	(28.3±1.8)^ijk^	(3.45±0.04)^m^	(8.0±0.0)^ef^	(1.4±0.4)^fghij^	(5.8±0.8)^efghij^	(53.9±4.6)^abc^	(82.6±5.3)^efg^	(23.7±2.7)^l^	(3.6±0.1)^cde^	(4.4±0.3)^d^	(2.37±0.02)^def^	(1.9±0.1)^g^
SCSH 15-19	(201.0±9.6)^b^	(43.9±1.8)^ab^	(3.79±0.03)^efg^	(8.0±0.0)^ef^	(1.9±0.4)^ab^	(4.3±0.8)^kl^	(42.13±4.02)^hi^	(76.0±6.9)^efghi^	(24.3±2.8)^kl^	(3.4±0.2)^def^	(4.4±0.2)^d^	(2.1±0.2)^hi^	(2.2±0.1)^ef^
SCSH 17-9	(103.3±11.5)^cde^	(26.7±2.3)^jkl^	(3.75±0.04)^fgh^	(8.0±0.0)^ef^	(1.3±0.1)^ghijkl^	(6.0±0.5)^efgh^	(58.1±4.6)^a^	(51.4±4.3)^jklm^	(37.8±3.6)^efghij^	(2.0±0.1)^jk^	(3.6±0.1)^gh^	(2.2±0.1)^fgh^	(1.3±0.1)^ij^
SCSH 19-2	(136.7±13.2)^cd^	(29.2±1.4)^hijk^	(3.55±0.03)^l^	(9.3±1.2)^bc^	(1.89±0.02)^abc^	(4.9±0.6)^hijkl^	(55.5±4.7)^ab^	(61.8±5.4)^jkl^	(32.0±3.1)^ghijkl^	(2.2±0.4)^ijk^	(3.1±0.1)^k^	(2.45±0.05)^cde^	(0.59±0.01)^l^
SCSH 19-6	(103.33±9.07)^fgh^	(36.6±2.8)^cdefg^	(3.72±0.01)^ghi^	(8.7±0.6)^cde^	(1.22±0.05)^ijkl^	(7.1±0.6)^cde^	(44.4±2.2)^efghi^	(74.3±6.9)^fghi^	(29.3±2.4)^ijkl^	(2.6±0.1)^hij^	(4.8±0.3)^c^	(2.2±0.1)^fgh^	(2.5±0.2)^cd^
SCSH 19-8	(130.0±12.0)^cde^	(29.8±3.1)^hijk^	(3.64±0.03^jk^	(7.3±0.6)^f^	(1.5±0.2)^defghi^	(4.8±0.5)^ijkl^	(50.7±3.6)^bcde^	(82.9±3.6)^ef^	(42.7±0.6)^cdefgh^	(2.5±0.2)^hijk^	(3.31±0.02)^ijk^	(2.14±0.01)^h^	(1.12±0.01)^jk^
SCSH 21-10	(130.0±5.0)^cde^	(31.6±1.2)^ghij^	(3.55±0.03)^l^	(8.0±0.0)^ef^	(1.9±0.1)^ab^	(4.1±0.1)^l^	(51.3±4.4)^bcd^	(75.4±6.9)^efghi^	(41.3±4.1)^defghi^	(2.9±0.1)^efgh^	(3.9±0.1)^ef^	(2.14±0.01)^h^	(1.8±0.1)^gh^
CRH 20-11	(85.0±5.0)^hij^	(44.7±3.8)^ab^	(3.68±0.04)^hij^	(10.0±0)^b^	(1.3±0.1)^hijkl^	(7.8±0.6)^bc^	(53.60±3.02)^abc^	(84.7±6.6)^def^	(44.3±1.2)^bcdefg^	(2.0±0.2)^jk^	(3.8±0.2)^fg^	(2.8±0.1)^b^	(1.02±0.01)^k^
Parentage													
Mosambi	(133.3±7.6)^cde^	(47.0±1.2)^a^	(4.73±0.03)^a^	(11.0±1.0)^a^	(1.5±0.1)^efghij^	(7.6±0.8)^bcd^	(58.4±0.8)^a^	(116.7±7.6)^b^	(36.5±3.5)^fghijk^	(1.8±0.2)^k^	(4.4±0.3)^d^	(2.9±0.2)^a^	(1.4±0.1)^i^
PS-2	(115.0±10.0)^def^	(26.6±2.0)^jkl^	(4.24±0.04)^b^	(9.0±1.0)^cd^	(1.5±0.1)^defghi^	(5.9±0.2)^efghi^	(42.0±0.7)^hi^	(168.2±14.1)^a^	(49.5±4.3)^abcde^	(4.1±0.9)^bc^	(3.9±0.1)^f^	(1.33±0.04)^l^	(2.50±0.04)^cd^
PS-5	(116.7±10.2)^def^	(20.7±1.5)^lm^	(3.93±0.03)^d^	(8.7±1.2)^cde^	(1.5±0.1)^efghij^	(5.9±0.2)^efghi^	(41.3±1.2)^hi^	(102.3±5.5)^c^	(50.8±2.9)^abcd^	(2.5±0.2)^hij^	(5.0±0.4)^c^	(2.2±0.1)^gh^	(2.7±0.4)^bc^
PS-10	(65.7±2.1)^jk^	(15.3±0.2)^m^	(4.08±0.02)^c^	(8.0±0.0)^ef^	(1.01±0.01)^l^	(7.9±0.1)^bc^	(44.0±1.6)^fghi^	(96.8±8.7)^cd^	(56.3±4.7)^ab^	(2.6±0.3)^ghij^	(4.2±0.1)^de^	(1.80±0.02)^jk^	(2.3±0.1)^de^
LSD (p≤0.05)	24.75	6.20	0.07	0.95	0.33	1.25	6.61	12.41	12.60	0.68	0.25	0.15	0.25

The results are presented as mean value±standard deviation (*N*=3). Mean values with different letters in superscript in the same column are statistically different (p≤0.05). JR=juice recovery, TSS=total soluble solids, TA=titratable acidity, AA=ascorbic acid, TPC=total phenolic content, TFC=total flavonoid content, DPPH=total antioxidants, TS=total sugars, RS=reducing sugars, NRS=non-reducing sugars

**Table S3:** Details of acidity specific markers showed 87.5 % polymorphism

Marker ID	Forward/reverse primer	F *t*_m_/°C	R *t*_m_/°C	*t*_a_/°C	Expected product size/bp	Observed product size/bp	Polymorphism
NRCH1	AACCTATGTCCCCCATGTGC TGCACCATTTTCTTTGGCCG	59.3	57.3	55	183	nd	Not amplified
NRCH2	AAGGGCTAAGGTTGGTGTGG CCCGTTTACATTGTCCTTCACC	59.3	60.3	55	148	100–120	Polymorphic
NRCH3	CGCAGCTGTAAACGATGACC CAGAGTCGGACTGATCCTGC	59.3	61.4	55	107	100–120	Polymorphic
NRCH4	AAGGGCTAAGGTTGGTGTGG CCCGTTTACATTGTCCTTCACC	59.3	60.3	55	148	100–120	Polymorphic
NRCH5	AACCTATGTCCCCCATGTGC CAGAGTCGGACTGATCCTGC	59.3	61.4	55	126	130–150	Polymorphic
NRCH6	AAGGGCTAAGGTTGGTGTGG CCCGTTTACATTGTCCTTCACC	59.3	60.3	55	148	150–190	Polymorphic
NRCH7	CGCAGCTGTAAACGATGACC CAGAGTCGGACTGATCCTGC	59.3	61.4	55	107	100–130	Polymorphic
NRCH8	AAGGGCTAAGGTTGGTGTGG CCCGTTTACATTGTCCTTCACC	59.3	60.3	55	148	110–190	Polymorphic

F=forward primer, R=reverse primer, *t*_m_ and *t*_a_=melting and annealing temperatures, nd=not determined

**Table S4:** Grouping of citrus fruit hybrids based on acidity-specific markers (three cross combinations)

Parent	Genotype	TA/%
PS-2 (pummelo red) × sweet orange cv. Mosambi	SCSH 3-14	1.56
	SCSH 3-15	1.72
	SCSH 5-5	1.79
	SCSH 9-20	1.64
	SCSH 3-10	1.37
	SCSH 9-2	1.35
	SCSH 13-14	1.07
	CRH 20-11	1.28
PS-5 (pummelo white) × sweet orange cv. Mosambi	SCSH 11-15	2.11
	SCSH 11-19	1.83
	SCSH 13-4	1.96
	SCSH 13-13	1.81
	SCSH 15-19	1.92
	SCSH 19-2	1.89
	SCSH 21-10	1.96
PS-10 (pummelo white) × sweet orange cv. Mosambi	SCSH 19-8	1.55
	SCSH 7-12	1.00
	SCSH 9-10	1.45
	SCSH 13-17	1.16
	SCSH 15-2	1.33
	SCSH 15-3	1.46
	SCSH 15-18	1.45
	SCSH 17-9	1.33
	SCSH 19-6	1.22

TA=titratable acidity

## References

[1] The UNICEF/WHO/WB Joint Child Malnutrition Estimates (JME) group released new data for 2021.Geneva, Switzerland: World Health Organization; 2021. Available from: https://www.who.int/news/item/06-05-2021-the-unicef-who-wb-joint-child-malnutrition-estimates-group-released-new-data-for-2021.

[2] 2021 Report of the Secretary-General on the work of the Organization. United Nations report. Geneva, Switzerland: United Nations; 2021. Available from: https://www.un.org/annualreport/2021/index.html.

[3] Yadava DK Hossain F Mohapatra T. Nutritional security through crop biofortification in India: Status & future prospects. Indian J Med Res. 2018;148(5):621. 10.4103/ijmr.IJMR_1893_18 30666987 PMC6366255

[4] Crops and livestock products. Rome, Italy: Food and Agriculture Organization of the United Nations; 2023[Accessed 7 August 2023]. Available from: http://www.fao.org/faostat/en/#data/QC.

[5] Raghavan S Gurunathan J. Citrus species– A golden treasure box of metabolites that is beneficial against disorders. J Herb Med. 2021;28:100438. 10.1016/j.hermed.2021.100438

[6] Paul DK Shaha RK. Citrus fruits in the northern region of Bangladesh. Pak J Biol Sci. 2004;7(2):238–42. 10.3923/pjbs.2004.238.242

[7] Okwu DE Emenike IN. Nutritive value and mineral content of different varieties of citrus fruits. J Food Technol. 2007;5(2):105–8.

[8] Bellavite P, Donzelli A. Hesperidin and SARS-CoV-2: New light on the healthy function of citrus fruits. Antioxidants. 2020;13;9(8):742. 10.3390/antiox9080742 10.3390/antiox9080742 PMC7465267 32823497

[9] Abobatta WF. Nutritional benefits of citrus fruits. Am J Biomed Sci Res. 2019;3(4):303–6. 10.34297/AJBSR.2019.03.000681

[10] Mahmoud AM, Ashour MB, Abdel-Moneim A, Ahmed OM. Hesperidin and naringin attenuate hyperglycemia-mediated oxidative stress and proinflammatory cytokine production in high fat fed/streptozotocin-induced type 2 diabetic rats. J Diabetes Complicat. 2012;1;26(6):483-90. 10.1016/j.jdiacomp.2012.06.001 10.1016/j.jdiacomp.2012.06.001 22809898

[11] Raveh E, Goldenberg L, Porat R, Carmi N, Gentile A, La Malfa S. Conventional breeding of cultivated Citrus varieties. In: Gentile A, La Malfa S, Deng Z, editors. The citrus genome. Berlin, Germany: Springer; 2020. pp. 33-48. 10.1007/978-3-030-15308-3_4 10.1007/978-3-030-15308-3_4

[12] Official Method AOAC. 947.05. Titrimetric method. Rockville, MD, USA: AOAC International; 2000.

[13] Official Method AOAC. 967.21. Ascorbic acid in vitamin preparations and juices. Rockville, MD, USA: AOAC International; 2000.

[14] Ranganna S, editor. Handbook of analysis and quality control for fruit and vegetable products. New York, NY, USA: McGraw-Hill Education; 1986.

[15] Zhishen J Mengcheng T Jianming W. The determination of flavonoid contents in mulberry and their scavenging effects on superoxide radicals. Food Chem. 1999;64(4):555–9. 10.1016/S0308-8146(98)00102-2

[16] Singleton VL Orthofer R Lamuela-Raventós RM. Analysis of total phenols and other oxidation substrates and antioxidants by means of Folin-Ciocalteu reagent. Methods in Enzymology. 1999;299:152–78. 10.1016/S0076-6879(99)99017-1

[17] Brand-Williams W Cuvelier ME Berset CL. Use of a free radical method to evaluate antioxidant activity. LWT - Food Sci Technol. 1995;28(1):25–30. 10.1016/S0023-6438(95)80008-5

[18] Lane JH, Eynon L. Determination of reducing sugars by Fehling's solution with methylene blue indicator. London, UK: N. Rodger; 1934.

[19] Jellinick G. Sensory evaluation of food theory and practices. Chichester, UK: Ellis Hardwood; 1985.

[20] Doyle JJ Doyle JL. A rapid total DNA preparation procedure from fresh plant tissue. Focus. 1987;12:13–5.

[21] Nucleotide [Internet]. Bethesda, MD, USA: National Library of Medicine (US), National Center for Biotechnology Information; [Cited 2023 Aug 02]. Available from: https://www.ncbi.nlm.nih.gov/nuccore/?term=citrus+sinensis+acidity.

[22] Du L Zhang C Liu Q Zhang X Yue B. Krait: An ultrafast tool for genome-wide survey of microsatellites and primer design. Bioinformatics. 2018;34(4):681–3. 10.1093/bioinformatics/btx665 29048524

[23] Rohlf FJ. NTSYSpc: Numerical taxonomy and multivariate system, v. 2.1. Port Jefferson, NY, USA: Applied Biostatics Inc.; 2000.

[24] SAS. v. 9.3, SAS Inc., Cary, NC, USA; 2011. Available from: https://support.sas.com/software/93/.

[25] R Core Team_R: A language and environment for statistical computing. Vienna, Austria: R Foundation for Statistical Computing: 2023.

[26] Dice LR. Measures of the amount of ecologic association between species. Ecology. 1945;26(3):297–302. 10.2307/1932409

[27] Devarajan R Jayaraman JK Somasundaram SM Ragupathy S Raman P Sathiamoorthy K Genetic diversity in fresh fruit pulp mineral profile of 100 Indian *Musa* accessions. Food Chem. 2021;361:130080. 10.1016/j.foodchem.2021.130080 34029894

[28] Fiévet JB Nidelet T Dillmann C De Vienne D. Heterosis is a systemic property emerging from non-linear genotype-phenotype relationships: Evidence from *in vitro* genetics and computer simulations. Front Genet. 2018;9:159. 10.3389/fgene.2018.00159 29868111 PMC5968397

[29] Rehman SU Abbasi KS Qayyum A Jahangir M Sohail A Nisa S Comparative analysis of citrus fruits for nutraceutical properties. Food Sci Technol. 2019;40 Suppl.:153–7. 10.1590/fst.07519

[30] Paithankar DH Nagre P Megha YVI Jadhao GG. Evaluation of acid lime clones for growth yield and quality. Int J Curr Microbiol Appl Sci. 2018;6:2737–45.

[31] Sharma A, editor. A text book of food science and technology. Lucknow, India: International book distributing Co.; 2006.

[32] Thakkar SR Shah PU. Sensory evaluation of dehydrated onion compared to fresh onion samples. Int Res J. 2009;2(7):57–60.

[33] Manay NS, Swamy S, editors. Food facts and principles. New Delhi, India: New Age International. Ltd Publishers; 2002.

[34] Feria-Morales A. Examining the case of green coffee to illustrate the limitations of grading systems/expert tasters in sensory evaluation for quality control. Food Qual Prefer. 2002;13(6):355–67. 10.1016/S0950-3293(02)00028-9

[35] Rowe D. Overview of flavor and fragrance materials. In: Goodner K, Rouseff R, editors. Practical analysis of flavor and fragrance materials. Chichester, UK: John Wiley & Sons Ltd.; 2011. pp. 1-22. 10.1002/9781444343137.ch1 10.1002/9781444343137.ch1

[36] Lego A Upadhyay S Bhutia KD Sharma L. Physico-chemical characteristics and sensory evaluation of Sikkim mandarin wine. J Postharv Technol. 2023;11(1):54–65.

[37] Idangodage IP De Siva AB Herath HM Jayasinghe JM. Development and physico-chemical evaluation of an isotonic *Nas Narang* (*Citrus madurensis*) sports drink. J Adv Food Sci Technol. 2023;6:75–85. 10.56557/jafsat/2023/v10i28130

[38] Mishra PK Ram RB Kumar N. Genetic variability, heritability, and genetic advance in strawberry (*Fragaria* × *ananassa* Duch.). Turk J Agric For. 2015;39(3):451–8. 10.3906/tar-1408-99

[39] García-Lor A Luro F Navarro L Ollitrault P. Comparative use of InDel and SSR markers in deciphering the interspecific structure of cultivated citrus genetic diversity: A perspective for genetic association studies. Mol Genet Genomics. 2012;287:77–94. 10.1007/s00438-011-0658-4 22160318

[40] Wu GA Terol J Ibanez V López-García A Pérez-Román E Borredá C Genomics of the origin and evolution of citrus. Nature. 2018;554:311–6. 10.1038/nature25447 29414943

[41] Panse VG. Genetics of quantitative characters in relation to plant breeding. Indian J Genet. 1957;17:318–28.

[42] Karmakar P Munshi AD Behera TK Kumar R Sureja AK Kaur C Quantification and inheritance of antioxidant properties and mineral content in ridge gourd (*Luffa acutangula*). Agric Res. 2013;2:222–8. 10.1007/s40003-013-0070-x

[43] Baswal AK Rattanpal HS Gill KS Sidhu GS. Genetic variability, heritability and genetic advance in grape fruit (*Citrus paradisi*) genotypes. HortFlora Res Spectr. 2016;5(3):228–32.

[44] Singh G Rattanpal HS Gupta M Sidhu GS. Genetic variability and heritability among mandarin (*Citrus reticulata* Blanco) genotypes under Indian sub-tropical conditions. Appl Ecol Environ Res. 2022;20(4):2913–30. 10.15666/aeer/2004_29132930

[45] Bastianel M De Oliveira AC Cristofani M Filho OG Freitas-Astúa J Rodrigues V Inheritance and heritability of resistance to *Citrus Leprosis.* Phytopathology. 2006;96(10):1092–6. 10.1094/PHYTO-96-1092 18943497

[46] Chen C Cancalon P Haun C Gmitter F. Characterization of furanocoumarin profile and inheritance toward selection of low furanocoumarin seedless grapefruit cultivars. J Am Soc Hortic Sci. 2011;136(5):358–63. 10.21273/JASHS.136.5.358

[47] Rajae Y Najat H Mohamed I Tarik A Ennacir H Adnane H Study of genetic variability, heritability and repeatability for fruit quality characters in *Citrus sinensis.* Plant Cell Biotechnol Mol Biol. 2019;20(19-20):860–8.

[48] Angami T Kalita H Lungmuana LT Makdoh B Bagra G Singh R Varietal assessment and genetic variability studies of pummelo (*Citrus grandis* (L.) Osbeck) accessions under mid hill conditions. Environ Ecol. 2022;40(3):1046–52.

[49] Colón AM Sengupta N Rhodes D Dudareva N Morgan J. A kinetic model describes metabolic response to perturbations and distribution of flux control in the benzenoid network of *Petunia hybrida.* Plant J. 2010;62(1):64–76. 10.1111/j.1365-313X.2010.04127.x 20070567

[50] Labroo MR Studer AJ Rutkoski JE. Heterosis and hybrid crop breeding: A multidisciplinary review. Front Genet. 2021;12:643761. 10.3389/fgene.2021.643761 33719351 PMC7943638

[51] Dubey AK Kholia A Sharma N Sharma RM. Assessing genetic diversity in Indian pummelo collections utilizing quantitative traits and simple sequence repeat markers (SSRs). Indian J Hortic. 2021;78(1):3–8. 10.5958/0974-0112.2021.00001.3

[52] Raigón MD Prohens J Muñoz-Falcón JE Nuez F. Comparison of eggplant landraces and commercial varieties for fruit content of phenolics, minerals, dry matter and protein. J Food Compos Anal. 2008;21(5):370–6. 10.1016/j.jfca.2008.03.006

[53] Nagesh VR Usharani G Reddy TD. Heterosis studies for grain iron and zinc content in rice (*Oryza sativa* L.). Ann Biol Res. 2012;3(1):179–84.

[54] Bell MA Travis MP. Hybridization, transgressive segregation, genetic covariation, and adaptive radiation. Trends Ecol Evol. 2005;20(7):358–61. 10.1016/j.tree.2005.04.021 16701394

[55] Goh RM Pua A Luro F Ee KH Huang Y Marchi E Distinguishing citrus varieties based on genetic and compositional analyses. PLoS ONE. 2022;17(4):e0267007. 10.1371/journal.pone.0267007 35436309 PMC9015143

[56] Drogoudi PD Vemmos S Pantelidis G Petri E Tzoutzoukou C Karayiannis I. Physical characters and antioxidant, sugar, and mineral nutrient contents in fruit from 29 apricot (*Prunus armeniaca* L.) cultivars and hybrids. J Agric Food Chem. 2008;56(22):10754–60. 10.1021/jf801995x 18975966

[57] Singh N Sharma RM Dubey AK Awasthi OP Porat R Saha S Harvesting maturity assessment of newly developed citrus hybrids (*Citrus maxima* Merr. × *Citrus sinensis* (L.) Osbeck) for optimum juice quality. Plants. 2023;12(23):3978. 10.3390/plants12233978 38068614 PMC10708354

[58] Merrick L, Meade K, Campbell A, Muenchrath D, Fei SZ, Beavis W. Inheritance of quantitative traits. In: Suza W, Lamkey K, editors. Crop Genetics. Ames, IA, USA: Iowa State University Digital Press; 2023.

